# Supplementary material for: DMRforPairs: identifying Differentially Methylated Regions between unique samples using array based methylation profiles
Source: BMC Bioinformatics. 2014 May 15;15:141. doi: 10.1186/1471-2105-15-141 (PMC4046028; doi:10.1186/1471-2105-15-141)
Supplement: Additional file 2 — DMRforPairs output for the comparison of A431-MCF7 and NA17018-NA17105. Please start from the HTML files in each folder. Available via the BMC Bioinformatics website. [file 1471-2105-15-141-S2.zip › 1394847754114233_MOESM2_ESM/A431_MCF7/figures/25230.pdf]

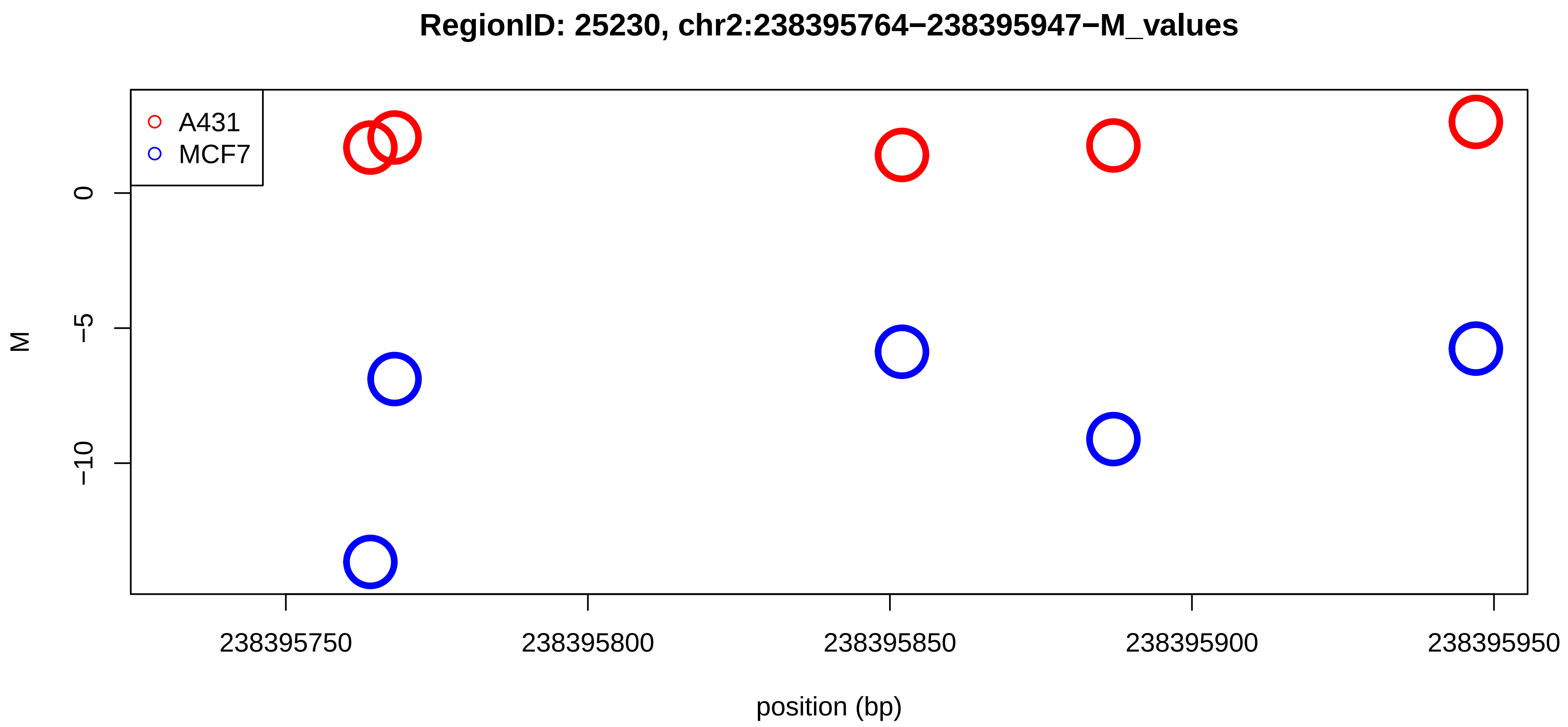
RegionID: 25230, chr2:238395764−238395947−M_values
A431
MCF7
0
−5
−10
M
238395750
238395800
238395850
238395900
238395950
position (bp)

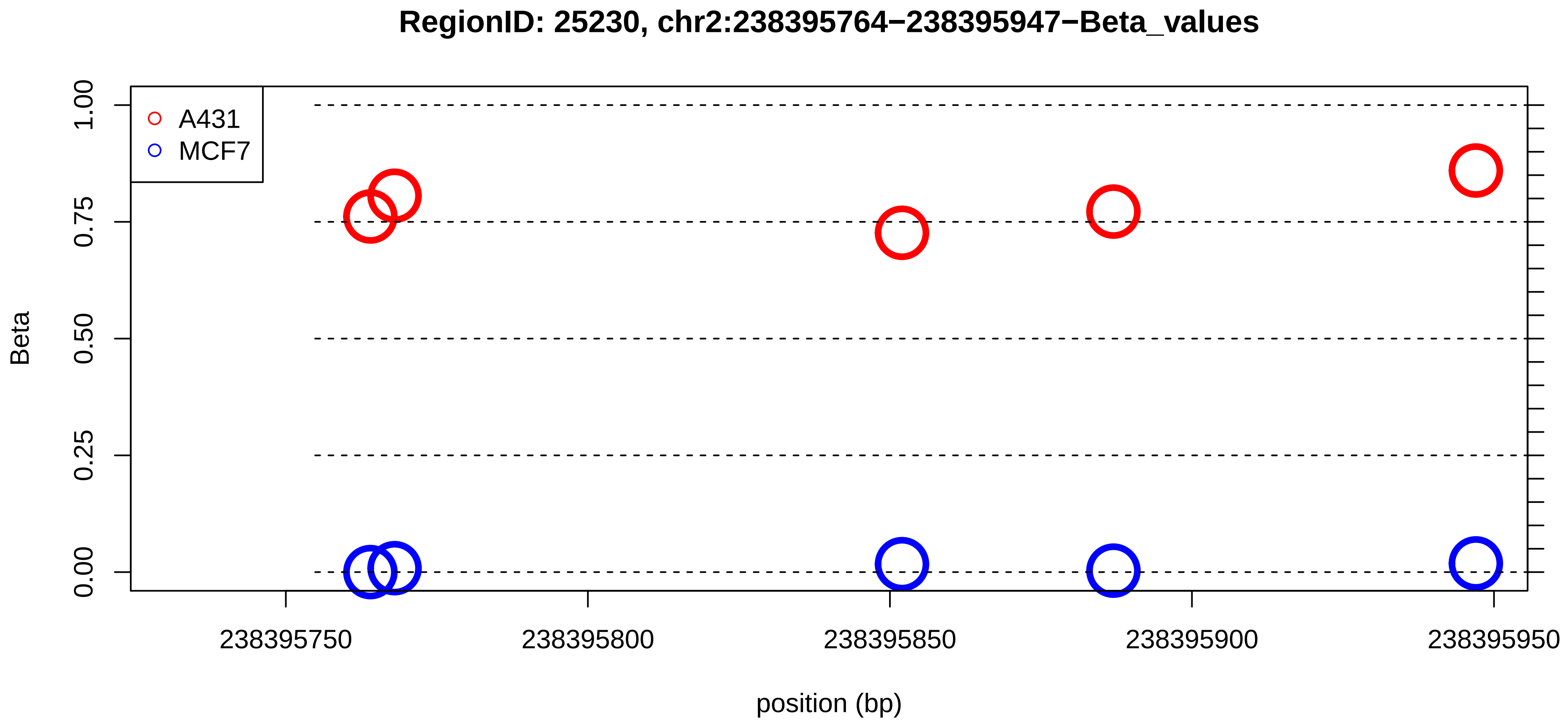
RegionID: 25230, chr2:238395764−238395947−Beta_values
A431
MCF7
1.00
0.75
0.50
0.25
0.00
Beta
238395750
238395800
238395850
238395900
238395950
position (bp)

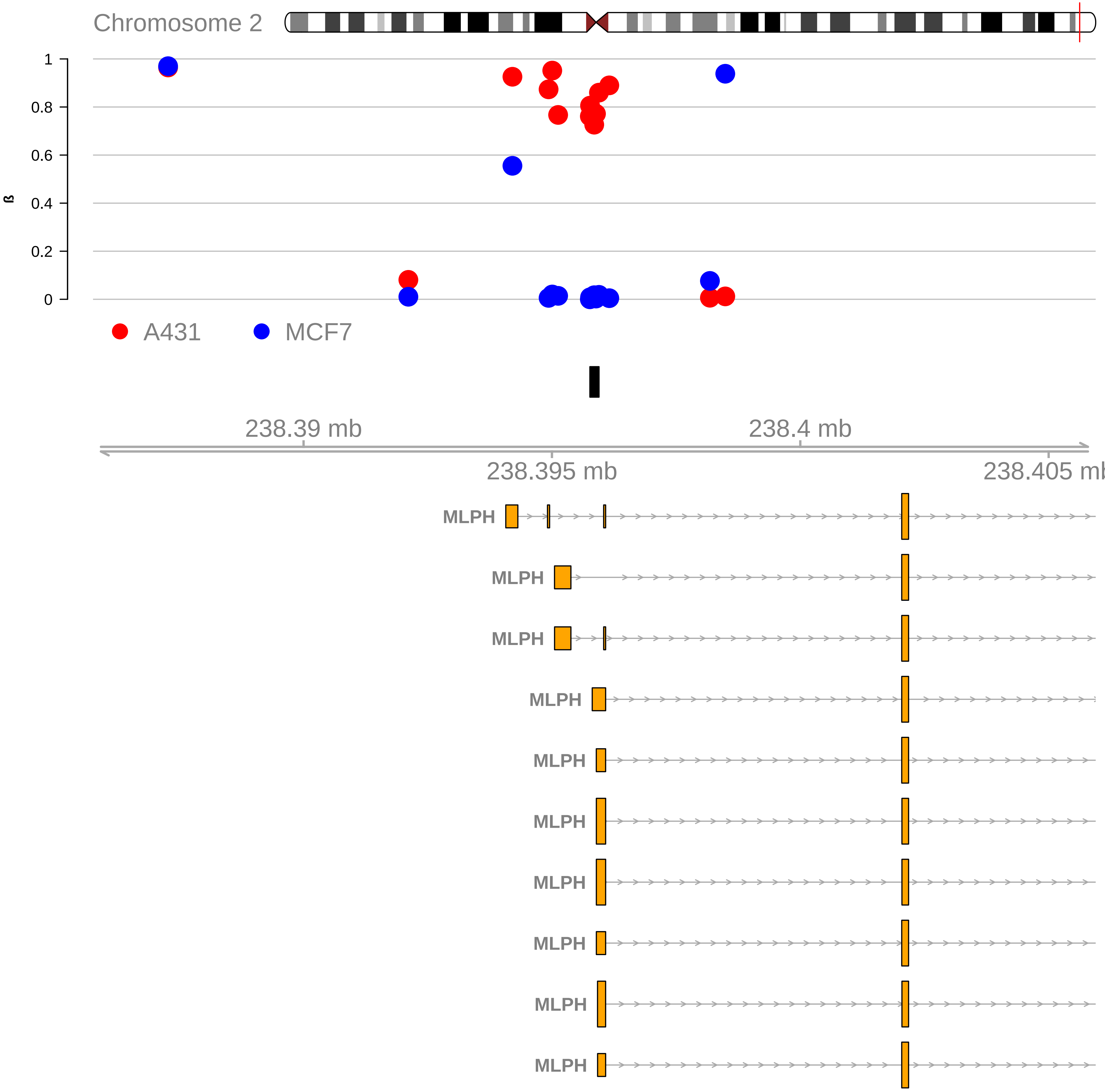

Chromosome 2
1
0.8
0.6
0.4
0.2
0
β
A431
MCF7
238.39 mb
238.4 mb
238.395 mb
238.405 mb
MLPH
MLPH
MLPH
MLPH
MLPH
MLPH
MLPH
MLPH
MLPH
MLPH
